# Ozonation as a Method of Abiotic Elicitation Improving the Health-Promoting Properties of Plant Products—A Review

**DOI:** 10.3390/molecules25102416

**Published:** 2020-05-22

**Authors:** Monika Sachadyn-Król, Sofia Agriopoulou

**Affiliations:** 1 Department of Chemistry, Faculty of Food Sciences and Biotechnology, University of Life Sciences in Lublin, 20950 Lublin, Poland; 2 Department of Food Science and Technology, University of the Peloponnese, 24100 Antikalamos, Kalamata, Greece; sagriopoulou@gmail.com

**Keywords:** ozone, elicitation, antioxidant activity, phenolics, vitamin C, anthocyanins, color, fruits, vegetables

## Abstract

In this review, the primary objective was to systematize knowledge about the possibility of improving the health-promoting properties of raw plant products, defined as an increase in the content of bioactive compounds, by using ozone. The greatest attention has been paid to the postharvest treatment of plant raw materials with ozone because of its widespread use. The effect of this treatment on the health-promoting properties depends on the following different factors: type and variety of the fruit or vegetable, form and method of ozone treatment, and dosage of ozone. It seems that ozone applied in the form of ozonated water works more gently than in gaseous form. Relatively high concentration and long contact time used simultaneously might result in increased oxidative stress which leads to the degradation of quality. The majority of the literature demonstrates the degradation of vitamin C and deterioration of color after treatment with ozone. Unfortunately, it is not clear if ozone can be used as an elicitor to improve the quality of the raw material. Most sources prove that the best results in increasing the content of bioactive components can be obtained by applying ozone at a relatively low concentration for a short time immediately after harvest.

## 1. Introduction

The expectations of modern consumers regarding their food focus not only on satisfying their hunger but also on the fulfillment of new, increasingly diverse, and individualized functions leading to a high quality of life. Daily consumption of fresh fruits and vegetables could significantly contribute to human health, as they are very rich in proven beneficial ingredients with antioxidant and medicinal action and could help prevent major diseases, such as cardiovascular diseases and certain cancers. The consumers expect that the food should support their health and help to maintain a good physical condition. On the one hand, consumers demand the highest quality food adapted to their individual needs, and on the other hand, they want food that is safe and healthy. This has led to the production of food from raw materials with high nutritional value and rich in bioactive substances. Previous studies have shown that regular consumption of fruits and vegetables can play an important role in protecting health by reducing the risk of various diseases [1,2]. Raw materials of plant origin and their extracts show effective anticancer properties [3,4,5], which can be attributed to the presence of phytochemicals, in particular, polyphenols, carotenoids, alkaloids, and nitrogen- and sulfur-containing compounds [6,7]. Polyphenols are a unique and important group of natural compounds [2,8,9]. Polyphenols can exert antitumor effect by inhibiting the growth of cancer cells or by changing the activity of intracellular enzymes during the stages of initiation, promotion, and progression of cancer [6,7,8,9]. Due to the aforementioned properties, the use of polyphenols for the enrichment of foods seems to be justified for the chemoprevention of cancer and other diseases. Plant extracts are used in place of synthetic antioxidants in the preparation of food [10,11] and they do not cause potentially toxic effects [12,13]. Another direction of research attempts to increase the bioactive compound content in raw plant materials by applying various techniques such as selecting suitable varieties [14], changing growing conditions [15,16], elicitation of various pathways [17], and improving methods of packaging and storage. Using edible coatings as carriers of pharmacologically active antioxidants also can result in additional improvement of the nutraceutical potential of the packaged food [18].

Ozonation is now widely used in the food-processing industry as a sanitizing agent and to extend the shelf life of the product, and thus increase production profitability by reducing trade losses. Globally, the use of ozone (trioxygen, O_3_) in the food-processing industry is at various stages of regulation in many countries, including the US, Japan, Australia, Canada, and the European Union [19]. Ozone rapidly degrades to oxygen and leaves no residue; thus, it is safe and the ozonated product is suitable for the certification of organic food [20]. The primary aim of ozonation is microbial decontamination. Ozone is mainly used in the decontamination of the food products, surface disinfection of equipment, decontamination of meat and meat products, poultry and eggs, seafood, fruits, vegetables, as well as dry foods [21]. It is used either as a gas or after dissolving in water [22]. Ozonated water is increasingly used in the fruit and vegetable processing industry for purifying fresh raw materials and extending their durability. Ozone has a broad spectrum of antimicrobial activity and is an effective agent in eliminating fungi, yeasts [23], and pathogenic bacteria such as *Escherichia coli, Salmonella typhimurium,* and *Listeria monocytogenes* [24,25]. Due to the mechanism of action of ozone, which acts via cellular lysis, microorganisms cannot acquire resistance to it. It effectively destroys even those microorganisms and spores that are resistant to chlorine-based disinfectants [26]. Ozone acts via attacking numerous cellular components, such as proteins, unsaturated lipids and respiratory enzymes in cell membranes, peptidoglycans in the cell envelope, enzymes and nucleic acids in the cytoplasm, proteins and peptidoglycans in spore mantles and virus capsids [27]. Non-heat-treated products usually contain various kinds of microorganisms (e.g., yeast, mold, and lactic acid bacteria) that cause deterioration of the product during storage. Ozone extends the shelf life of fruits and vegetables by destroying the microorganisms and by blocking the action of ethylene, thereby delaying the aging process of plant raw material [28]. 

However, it soon became apparent that ozone treatment may have additional benefits such as effective degradation of pesticides [29,30,31] and mycotoxins [32,33,34,35]. In addition, it also prevents the growth of mycotoxins during storage [34,36]. It has been reported that ozone also degrades water-soluble pesticides, thereby limiting the possibility of subsequent infection with dissolved pesticides in water for reuse [30]. The high oxidative nature of ozone or the radicals generated during the process of ozonation cause molecular degradation of pesticide residues [37]. However, some degradation products were found to be more toxic than the parent compound [38].

Recent research indicates that ozonation can be used as an abiotic elicitor of plant defense mechanisms in low-processed food products of plant origin [39,40,41,42]. It can enhance the content of secondary metabolites and antioxidant activity. The development of methods increasing the content of antioxidant substances from their respective sources—to improve their purity and quality as well as their sustainability—is a key subject. Despite the known mechanisms of action of ozone, there are still many unknowns, especially regarding its action on different food categories. Depending on its origin, dry matter content or the fat content of the food, ozone can have a different effect on the final quality of the product. Many studies have been published to this day, and some contradict the others. Therefore, the purpose of this review was to systematize knowledge about the possibility of improving by ozone the health-promoting properties of raw plant products defined mainly as the increasing in the content of bioactive compound. 

## 2. Elicitation

Plants synthesize many biologically active compounds, which are called secondary metabolites. These metabolites are species-specific and are not involved in the processes of general metabolism but are essential in adapting plants to changing environmental conditions. So far, several secondary metabolites have been reported, such as alkaloids, phenolic compounds, essential oils, tannins, sterols, phytoalexins, terpenes, and coumarins [43]. Currently, techniques to increase the content of bioactive compounds that have documented pro-health effects are being sought. Products containing genetically modified organisms (GMO) are not socially trusted; therefore, elicitation has recently become a more popular method to enhance the quality of edible plants, especially in relation to their health-promoting properties [44]. Elicitation is a stress factor that triggers the production of secondary metabolites, such as antioxidants and other metabolites. Elicitors can be abiotic (substances of nonbiological origin and physical factors such as pH, the concentration of heavy metals, salt stress, water stress, light, and temperature) or biotic (substances from a biological origin, either from pathogens or by the plant itself) [45,46]. Due to the fact that abiotic or biotic stresses increase the production of bioactive components, it was assumed that they can improve the biological activity food of plant origin which thus becomes the functional food. 

To date, various elicitors have been used in plant cell culture to increase the production of secondary metabolites, mainly for pharmaceutical purposes. Preharvest elicitation has been implemented as seed priming, soaking seeds in a water-elicitor solution, after seedling through exogenous spraying treatment, or in a hydroponic system [45]. According to Złotek et al. [47], lettuce elicitation by jasmonic acid and arachidonic acid may enhance its health-promoting properties without losing its sensory quality. Another study [48] shows that preharvest treatment of butterhead lettuce with chitosan and tea tree essential oil enhanced the content of health-promoting phytochemicals, without affecting its organoleptic quality. Some studies have also shown that elicitation in edible sprouts elevates their antioxidant potential. Świeca and Baraniak [49] have shown that elicitation with H_2_O_2_ may influence the quantitative and qualitative profile of phenolic compounds in lentil sprouts. Gawlik-Dziki et al. [44] have shown that sprouting and elicitation of wheat are simple techniques of improving the bioactive content of the wheat flour. Natella et al. [50] have shown that total phenolic and flavonoid contents in broccoli sprouts were significantly affected by mannitol, sucrose, sodium chloride, 1-aminocyclopropane-l-carboxylic acid, and salicylic acid in a dose-dependent manner. Studies on the elicitation of herbs are also being conducted. Złotek et al. [51] studied the effect of elicitors on Origanum majorana L. According to their results, jasmonic acid elicitation significantly increased the carotenoid content and yeast extract increased the antioxidant content. Foliar application of methyl jasmonate and salicylic acid increased (2-fold) the concentration of folic acid in coriander foliage, and folates demonstrated higher stability during processing and storage [52]. Preharvest elicitation with methyl jasmonate might enhance the anthocyanin and flavonol content and increase the antiradical activity of black currant fruits [53]. The most popular abiotic elicitors are heavy metals, temperature, light, salinity, and drought [46]. Exposure of field-grown plants to the UV-B radiation increased the yield of essential oils and phenolic content [54]. An increase in salinity increased the polyphenol content in various plants [55].

So far, according to the available literature there is less research on post-harvest elicitation than those concerning pre-harvest. However, according to the available literature, peanut kernels stressed with UV light or ultrasound did not show an increase in the antioxidant activity and total phenolic content; however, these stresses were effective in increasing trans-resveratrol concentration. It is noteworthy that the effect of UV radiation depends on the intensity and the dose. The low intensity of UV-B irradiation and less number of doses delayed chlorophyll degradation and senescence; however, it did not improve the antioxidant capacity and phenolic content after long-term storage [56]. In contrast, high-intensity UV-B irradiation transiently increased the antioxidant capacity of broccoli florets 6 h after treatment [57]. Postharvest application of methyl jasmonate increased the antioxidant capacity of strawberry fruits. It also increased the concentration of total phenolics, anthocyanins compounds, and volatile chemicals up to 5–7 days of treatment. However, longer storage of methyl jasmonate-treated strawberry fruits resulted in the loss of fruit quality compared to the control sample [58].

## 3. Ozone as an Elicitor

Ozone can easily be generated and applied, and it can be easily removed at any time after application. Therefore, it is a convenient elicitor. There are two main types of ozonation: aqueous and gaseous phases. Aqueous ozone is obtained by introducing ozone gas generated by the ozonizer into the water. Trioxide with high oxidation and reduction potential is also well soluble in water, which can be used as a disinfectant. Another form is gaseous ozone, i.e., direct exposure of products to the tritylene produced by the generator. The raw materials are placed in a modified atmosphere enriched in ozone. Usually, products are periodically cooled with a specific ozone stream [39]. This method requires the use of devices intensifying the mass exchange between the disinfected product and the stream of gas produced. Due to the high toxicity of ozone, it is necessary to use closed devices during gas ozonation, which makes it more difficult than ozonation in water. It is proven that one hour human exposure to ozone concentrations of 2, 4, 15, and 95 ppm causes symptomatic, irritant, toxic, and irreversible lethal effects [21]. Most of the research has been focused on the issues related to the shelf life and overall acceptability of the food product, whereas the other has studied on the physiological characteristics such as loss of weight, texture, and color; physicochemical characteristics such as ascorbic acid content, total soluble solids, phenolic content, and antioxidant activity. The most commonly used technique of ozonation for plant products is long storage in an atmosphere containing a low concentration of ozone [59]. The second approach involves a single ozone treatment with a high dose. An interesting comparison of short- and long-term treatment has been made by Botondi [23], who showed that these two approaches can have a very different result. Ozone shock treatment (1.5 g/h for 18 h) preserved polyphenols, anthocyanins and carotenoids in wine grapes during postharvest dehydration. However, ozone long term treatment (additionally 0.5 g/h for 4 h each day until 35% weight loss) resulted in significant decrease in phenolic and anthocyanin contents. Wu et al. [60] concluded that a single ozone treatment (4 mg/m^3^ for 10 h) converts metabolic products from organic acids, soluble sugars, and alcohols in primary metabolites to volatile compounds, amino acids, and fatty acids. His study also showed that 102 metabolites showed differential accumulation after ozone treatment.

The effect of ozone on organic and inorganic compounds is the result of either a direct reaction of ozone or the reaction of radicals formed by its decomposition [27]. Ozone is a triatomic oxygen formed by a high energy input to oxygen, which decomposes and releases free hydroxyl (HO·), hydroxyperoxy (HO_2_·), and superoxide (O_2_·) radicals. Molecular ozone reactions are selective and are often limited to unsaturated aromatic and aliphatic compounds that are oxidized by cycloaddition of the O_3_ molecule for double bonding. O_3_ enters plants through open stomata and reacts with constituents of aqueous apoplast in the form of reactive oxygen species (ROS) [61]. Ozone interacts with the components of the plasma membrane’s ion and calcium channels, resulting in severe disruption of the membrane and immediate and massive production of ROS and certain enzymes such as superoxide dismutase and peroxidases [62]. This event induces many physiological changes in horticultural products, including changes in proteins and enzymes related to the plant defense mechanisms [63]. O_3_ can increase the plant’s stress resistance by stimulating the ROS-scavenging system in cells, which increases the synthesis of the antioxidant enzymes. Several genes are expected to be involved in the natural defense response in plants after ozone exposure: chitinase CHI1, β-glucanase GNS1, phenylalanine ammonia-lyase PAL1, peroxidase POD1 [64], and phosphorylase PR.

In general, ozonation as a method of elicitation might be applied at the stage of sowing seeds, planting, or during growth. Plant ozone defense responses depend on the interconnectedness between many complex signaling pathways and metabolic signals. The most important of them are abscisic acid, ethylene, jasmonic acid, and salicylic acid; however, other hormones might be involved in the defense response [65]. 

As shown in Table 1, this approach allows for the improvement of quality parameters of plant products; however, it has a major disadvantage: is quite difficult to carry out. It often requires specialized systems of ozone distribution and also, e.g., hydroponic fertilization or airtight chambers. In comparison with other methods of elicitation, pre-harvest treatment with ozone gas is expensive. Therefore, postharvest ozonation is more frequently used. 

The main problem of using ozone in a postharvest treatment is not only the proper selection of form, concentration, and time depending on the type and quantity, but also the initial quality of each particular commodity. The sensitivity of fruits and vegetables to ozone has been found to vary according to the type and species within a given type [71,72,73]. This might be the primary reason for the difference in response of plant products against different ozone treatment. It also seems that the final effect, in addition to those listed, depends also on the time from harvesting to ozonation. It is very difficult to compare effects especially in a situation where different conditions and different varieties within a species were used. Judging the effects of ozonation is further hampered by the fact that not all researchers provide the conditions, variations, and varieties in detail. However, below is an attempt to systematize knowledge in terms of phenolic content; flavonoid content; organic acid and vitamin C content; antioxidant activity; and color index in terms of the content of chlorophylls, carotenoids, or anthocyanins.

### 3.1. Phenolic Content

Phenolic compounds include oleuropein; hydroxybenzoic acid derivatives; cinnamates, isoflavones; lignans and stilbenes; anthocyanins; flavanones; chalcones and dihydrochalcones; flavonols, flavones, and flavonols; proanthocyanidins and tannin-like compounds; and ellagitannins and all their derivatives or related compounds. Caffeic acid derivatives, flavonols (mainly quercetin and its derivatives), catechins, isoflavones, flavanones, anthocyanins, and resveratrol are the most biologically active phenolic compounds [74]. In terms of the effects of ozonation on the phenolic, flavonoid, and organic acid contents, there is no single consensus among various groups of researchers. For instance, in terms of total phenolics content, there are 17 reports about the positive effect of ozonation, 16 about the negative effect, and 16 about no effect of ozone treatment on the aforementioned parameters (Table 2). After analyzing the available data, it can be concluded that the most important factor that is responsible for the difference in outcome is different concentrations of ozone and different methods of administration. According to the literature, too high doses are not beneficial, which may cause oxidation. For example, in the case of peach 200 mg O_3_/m^3^ for 60 days turned out to have negative effects. Similarly 5–20 g O_3_/dm^3^ in case of strawberry. In general, fruits are more sensitive to the oxidizing effects of ozone than vegetables. In many analyzed experiments on vegetables no changes were observed. The degradation in the quality of the ozone-treated food product might be attributed to two possible reactions: direct reaction of ozone with the target compound or its intermediates and the indirect reaction with the radicals generated through the decomposition of ozone [75,76]. The direct reaction is described in the literature as the Criegee mechanism, where ozone undergoes 1-3 dipolar cycloaddition with the double bond present, leading to the formation of ozonides from alkenes with aldehydes or ketone oxides as intermediates [77]. Oxidative disintegration of ozonide and formation of carbonyl compounds occurs, which in turn leads to the formation of carboxylic acids and ketones. This not only affects the color of the treated commodities but also alters the levels of ascorbic acid and total phenolics [78]. The form of ozone applied to the product is also very important. Ozonated water is very mildly reactive, and only a single publication shows the negative impact of ozone on the product [79]. In addition to the dose and form of ozone, the method of administration (one time, continuous, or repeatable) also matters. Short-term treatment with ozone (1.5 g/h for 18 h) can preserve the polyphenol and anthocyanin content in the wine grapes, whereas long-term treatment (until 35% weight loss) mostly decreases the polyphenol content [23]. 

There is a lot of discrepancies about the potential ozone elicitation of phenolic compounds. Two probable mechanisms are considered. The first hypothesis is that phenolic compounds are released through partial destruction of cell structure [80].

Ozonation causes changes in the skin structure and in the cell wall, which might free some of the conjugated phenolics, which in turn can lead to an increase in the extraction efficiency [81]. The sensitivity of phenolic compounds and other antioxidants to ozone is most probably affected by the type and location of the compound in the cell [61]. Depending on the structure, phenolic compounds exhibit different tendencies to accumulate within the cell wall [81]. Ozone stimulates the antioxidant systems mainly in the apoplast. Ranieri et al. [82] observed that ozone treatment increased free and glycoside-bound phenols in residual cell material; however, it greatly reduced the free phenolic content in intercellular washing fluid. Another study revealed that ozone treatment decreased the quantity of wall-bound phenolic compounds [83]. The second hypothesis is that phenolic compounds are increased due to the changes in enzyme concentration and their activity, which is induced by ozone. The majority of the articles have focused their research precisely on the second theory. Phenolic compounds can be rapidly accumulated due to the activation of preexisting enzymes [84]. Some studies demonstrated that the induction of phenylalanine ammonia lyase (PAL) by ozone is essential for the accumulation of different phenolic compounds in plants [74,85]. However, for example Sachadyn-Król et al. [86] observed lower activity of PAL and higher activity of oxidative enzymes responsible for quality loss due to phenolic degradation.

The literature describes that the decrease in the content of phenolic compounds is mainly due to the oxidizing potential of ozone. Phenolic compounds are particularly susceptible to ozone attack. The degradation may result from a variety of possible chemical reactions. For example, ozone can cleave the benzene ring of phenolic groups and phenolic compounds can undergo oxidation via Criegee mechanism [87]. Oxidation may occur through nucleophilic and electrophilic substitution reactions leading to the oxidation of organic compounds. Ozone favors the formation of hydroxylated compounds and quinones [88], which can cause the darkening of foods [89]. The auto-decomposition of triatomic oxygen is accompanied by the production of numerous ROS. ROS, in turn, can be scavenged by the phenolic compounds, which might reduce the levels of phenolic compounds after ozone exposure [78]. 

In the case of anthocyanins, five authors have observed an increase and all this observations were for soft berries with quite high initial anthocyanin content. In general, with the increase in the concentration of ozone and treatment time, the content of anthocyanin decreases. However, some studies have reported that anthocyanin content was not affected by ozone treatments, whereas the others have reported a statistically lower content in ozone-treated fruit juices than that of control samples (Table 2).

The literature provides contradictory results about the effects of ozone on organic acids. For example, ozone increased the concentration of hydroxycinnamic acid derivatives in table grapes [90], chicoric acid in chicory [91], and fumaric acid in tomato [92]. However, ozone decreased the content of chlorogenic acid, caffeic acid, and cinnamic acid in apple juice [76], hydroxycinnamic in white wine grapes [93] total organic acids in kiwifruits [94]. The content of ursolic acid in apple remained constant [95].

### 3.2. Vitamin C

Ascorbic acid is thermolabile, easily oxidizable, and highly sensitive to various processing and storage conditions [114]. Pignocchi et al. [156] postulated that apoplastic ascorbic acid represents the first line of defense against ozone. In addition, the activity of ascorbate oxidase had a direct effect on the level of apoplastic ascorbic acid. In this review article, the publications were also reviewed for the elicitation by ozone and vitamin C content of the product. Twenty-two sources provide information about the decrease in vitamin C content (measured mainly as ascorbic acid), 17 about the increase, and 15 about no changes after ozonation (Table 2). The response of plant tissues to various stresses such as ozone may affect the vitamin C regeneration capability. In general, nongreen tissues contain lower amounts of ascorbic acid than that of green ones [137]. Therefore, green fruits and vegetables show greater effects of oxidation due to ozone treatment. However, some studies conducted on green plant products showed that ozonation has no negative impact on ascorbic acid content in lettuce [146,147,149], spinach [61], and rocket leaves [150]. Setiasih et al. [105] exposed guava fruits for short time (5 min) and low concentration (1.1 ppm) and found that ozone did not penetrate the fruit, thereby preserving the vitamin C content. Venta et al. [153] also did not observe any effect of ozone treatment on the content of vitamin C in tomatoes. They treated the tomatoes with gaseous ozone and found that it did not penetrate through the surface of tomatoes. Ascorbic acid is water-soluble, and therefore it is present in rich quantities inside the tomatoes, which was prevented from degradation.

Ascorbic acid degradation can occur by both oxidative and nonoxidative mechanisms. It undergoes direct oxidation with ozone molecules or undergoes degradation due to the generation of free radical intermediates [61,114]. However, the majority of the occasions, it is degraded by the free radical mechanism [157]. Dehydroascorbic acid, an oxidation product of ascorbic acid, also exhibits biological activity as a vitamin. It can be reduced to form ascorbic acid or oxidized further to form diketogulonic acid, which exhibits no vitamin activity. The levels of the ascorbate oxidase, which oxidizes ascorbic acid to dehydroascorbic acid, increases under stress conditions, thereby promoting the degradation of ascorbic acid [61,158]. Rabie et al. [159] demonstrate that the scavenging of free radicals formed during the decomposition of ozone is responsible for the reduction of ascorbic acid content in the fruit juices. Activation of ascorbate oxidase under stress conditions is another factor that might contribute to the degradation of ascorbic acid to dehydroascorbic acid [73,78,158].

The authors who noticed the positive effect of ozonation on the vitamin C level explain this fact by the preservation of its content caused by stimulating the natural defense mechanisms of the plant. This might be clarified in terms of increased synthesis and accumulation of antioxidant compounds, such as ascorbic acid, in the cell to resist oxidative stress. Ozonized juices retained 68% more of vitamin C, whereas pasteurized samples preserved only 39% [111]. However, prolonged storage causes a decline in the content of ascorbic acid as the fruit condition deteriorates [100,112]. 

### 3.3. Antioxidant Capacity

The literature demonstrates a high correlation between total antioxidant capacity and phenolic content [152,160]. The observed changes in antioxidant activity could be associated with the content of ascorbic acid and/or phenolic compounds, presumably due to oxidative stress [143]. Antioxidant analysis obtained by different methods, such as the diphenyl-1-picrylhydrazyl (DPPH), ferric reducing antioxidant power (FRAP), oxygen radical absorbance capacity (ORAC), 2,2-azinobis(3-ehtylbenzothiazoline-6-sulfonic acid) diamonium salt (ABTS), 6-hydroxy-2,5,7,8-tetramethyl-chroman-2-carboxylic acid (Trolox)-equivalent antioxidant capacity (TEAC) assays have been found to be strongly correlated [161]. Wold et al. [162] postulated that color measurements can be used as a nondestructive method to assess total antioxidant capacity. They obtained a high correlation between values of L*, a*, b*, hue, and FRAP values in tomatoes.

Most authors agree that ozone increases the antioxidant activity of the product, some did not find any change, and some reported a decrease in the activity (Table 2). Miller et al. [115] for melon, García-Mateos et al. [88] for pitaya juice, Glowacz and Rees [143] for chili peppers treated with higher ozone concentration, Auguspole et al. [79] for shredded carrots and Karaca et al. [61] for parsley leaves observed a decrease in the antioxidant activity. A few more studies have shown that ozone treatment with concentrations up to 1 μmol/mol did not affect the antioxidant activities [59,150]. In general, an increase in the antioxidant activity appears to have a beneficial effect in the health-promoting quality of plant products.

Ozone treatment, antioxidant capacity and enzyme activity could have direct relationship in plants. O_3_ can cause increased production of ROS in the fruit cells, and consequently induce higher activity of antioxidant enzymes [64]. Primarily polyphenol oxidase (PPO) and peroxidase (POD) enzymes are directly related to oxidative stress. The PPO enzyme catalyses oxidation of hydroxyl groups in phenolic compounds, which leads to the browning. POD is directly involved in inactivation of ROS as well as processes of lignification and ethylene biosynthesis; POD also exhibits antioxidant properties. The activity of these enzymes in fruits and vegetables increases in the post-harvest period [86]. The main role of these enzymes is to protect the plant from oxidative stress and slowing down the aging process of fruits or vegetables. The authors present very different results regarding the effect of ozone on the activity of enzymes involved in defense reactions. For example, Sachadyn-Król et al. [86] noted an increase in the POD and PPO activity in pepper fruits in particular under the influence of the longer ozone-treatment period (3 h). In turn, Chauhan et al. [139] observed lower activity of the same enzymes in carrot samples treated with ozonated water compared to control samples. It appears that the use of inadequate long exposure time in combination with too high concentration of ozone might induce the accumulation of ROS which cause the enhanced oxidative stress and finally induce the senescence [122].

### 3.4. Color

Color is one of the most important distinguishing characteristics of a raw product that influences consumer choice. It is affected by the content of natural pigments such as chlorophylls, carotenoids, and anthocyanins, as well as other pigments resulting from enzymatic and nonenzymatic reactions [163]. For instance, flavonols, phloridzin, and hydroxycinnamic acids can contribute to changes in the color of apple juice [164] and carotenoids in orange juice [114]. Anthocyanins impart a red color to the fruits such as strawberries, red berries, cherries, table and wine grapes, red skinned apples. These pigments are also responsible for imparting the antioxidant properties [125]. Ozone affects the quality and quantity of these pigments. According to the literature, 18 sources show negative changes in the color parameter, 11 showed no changes, and 2 showed improvement (Table 2). Ummat et al. [136] showed no immediate effects of ozone on color of shredded green bell pepper; however, ozone-treated samples showed better retention of color for a longer time. Ozone-treated strawberry fruit maintained the best fruit color because of the higher amount of anthocyanins; however, they applied low concentration and short contact time (0.1 ppm for 2 min). Longer duration of ozonation could not retain better fruit color as compared to shorter duration. Similar to that of phenolic compounds, in the case of color preservation, ozonated water has been reported to be better than that of gaseous form. Bialka and Demirci [165] demonstrated that the color of blueberries subjected to gaseous ozone appeared darker, whereas after applying aqueous ozone, there was no significant change in color.

The effect of ozonation on the content of carotenoids is varied. Some studies demonstrate the positive effect of ozonation, some demonstrate its negative effects, and some could not prove that ozone affects the levels of carotenoids (Table 2). The decrease in the content of β-carotene and lycopene in fruits and vegetables subjected to high concentrations and long exposure to ozone might trigger the oxidative cleavage of carotenoids leading to the production of abscisic acid (ABA) [23]. However, the literature describes slightly different observations with respect to chlorophyll pigments. Most authors claim that ozone does not cause significant changes in chlorophyll levels. Chitravathi et al. [144] observed a positive effect of ozonation in inhibiting the degradation of chlorophyll during storage time. Ozone might inhibit the degradation of chlorophyll content and limit the activity of chlorophyll-degrading enzymes, such as chlorophyllase, chlorophyll-degrading peroxidase, Mg-dechelatase, and pheophytinase [166].

The strong oxidizing potential of ozone is derived from the detached oxygen atom. Ozone and the generated free radicals may open aromatic rings leading to the partial oxidation of products, such as organic acids, aldehydes, and ketones. The ring-opening step due to the formation of ozonide is the crucial step of degradation [87,167]. Breakdown of conjugated double bonds in chromophores of organic dyes, anthocyanins, and carotenoids by ozone causes color loss [76]. Ozone also forms other high-reactive species such as ·OH, HO_2_·, ·O_2_^−^, and ·O_3_^−^, which additionally contributes to increased degradation [130,155,167]. The color degradation seems to be a function of the concentration of ozone and treatment time. A previous study reported a slight darkening and a slight loss of color intensity in pitaya juice, which might be attributed to the high concentration and long treatment time [89]. Darkening is a common problem observed during juice processing, which is mainly caused by the activity of polyphenol oxidase. The reaction is often referred to as enzymatic browning. Nonenzymatic browning may in turn result from the Maillard reaction; however, caramelization and other reactions may also form products that are similar to those resulting from the Maillard reaction [114,168]. Some studies have demonstrated an inhibitory effect of ozone on enzymes responsible for browning, probably due to the high oxidation potential of ozone [142,148]. The time from the harvest to ozone treatment is very important, and the inhibitory effect is commonly observed only at the beginning of the storage period [169]. After this, browning greatly increases with increasing ozone concentration and storage time. This is probably caused by the byproducts formed from the decomposition of ozone over time leading to the browning of the product’s surface [163]. According to a previous study, the surface of the fruit gets damaged due to the physiological stress caused by the oxidation reaction with ozone [105,170]. Cullen et al. [171] have reported that ozone induces nonenzymatic browning by the oxidation of the phenolic compounds.

## 4. Summary

The effect of ozone treatment depends on many different factors, among which the most important are as follows: type and variety of fruits or vegetables being treated, form and method of ozone treatment, and dosage of ozone. Higher dosages and longer contact time might result in increased oxidative stress which degrades the quality of the fruit or vegetable. Biologically active compounds may be oxidized both via direct and/or via indirect reactions. In most of the articles reviewed, there is a decrease in the content of vitamin C and the deterioration of the color, which might be caused due to the degradation of chlorophyll and browning reactions. An increase in anthocyanin content in soft berries has been reported, and in general, this type of fruit is more susceptible to ozone elicitation. It seems like a lot of research has been done on the subject, unfortunately, among the reviewed articles, there is no clear indication as to the ability of ozone as an elicitor. Literature suggests that the best results are obtained by keeping a low concentration and short treatment time for ozone immediately after harvest. More research is needed on the comparison of different methods of ozone application as part of the same experiment along with an attempt to explain the mechanisms. From a more practical point of view, extensive studies on mathematical modeling of various ozonation parameters and their interdependence would also be highly desirable.

## Figures and Tables

**Table 1:** Effect of pre-harvest ozone treatment on the health-promoting qualitative features.

Product	Effect	Application Form	Ozone Dose	Process Duration	Source
Bobal Grapevines	+Terpenoids +Total anthocyanins	Water	NA	Single application 6 weeks before harvest	[66]
Broccoli plants	−Glucoraphanin +Glucobrassicin +Glucosinolates +Phenolics	Water (foliar application-spraying or root application)	0.2 mg/L	Foliar spraying on 2 consecutive days 2 times per week until harvesting (90 days) or 32 root treatments	[67]
Capsicum seedlings	±Total phenolics ±Vitamin C	Water (under greenhouse conditions)	0.4 ppm, 750 mV	NA	[68]
Passion fruit liana seeds	+Ascorbic acid +Carotenoids +Flavonoids	Gaseous	22, 41, and 58 ppb/h AOT_40_ and 13.52, 17.24, and 20.62 mmol/m^2^ POD_0_	over 97 days	[69]
Red pepper fruit from *Capsicum baccatum*	−Capsaicin +Total carotenoids +Total phenolics +Hydroxyl radical-scavenging activity	Gaseous	171.6 µg/m^3^	62 days	[70]
Strawberries (*Fragaria* x *ananassa* Duch.)	−Ascorbic acid ±Antioxidative capacity ±Anthocyanins ±Phenolics	Gaseous	156 μg/m³	8 h/day for 5 days a week for 2 months	[71]
Watermelon seedlings	+Total phenolic +Antioxidant activity ±Vitamin C	Water (under greenhouse conditions)	0.4 ppm, 750 mV	NA	[42]

NA—Not available, AOT_40_—Accumulated Ozone over Threshold of 40 ppb, POD_0_—Phytotoxic Ozone Dose.

**Table 2:** Effect of post-harvest ozone treatment on the health-promoting qualitative features.

Product	Effect	Application Form	Ozone Dose	Process Duration	Source
Apple	−Ascorbic acid −Cyanidin-3-glucoside	Water	200 mg/h	15 and 30 min	[96]
Apple	±Ursolic acid −Total flavonols ±Total phenols	Gaseous and water	NA	NA	[95]
Apple (fresh-cut)	±Total phenols +Antioxidant capacity	Water	1.4 mg/L	5 and 10 min	[97]
Apple juice	−color −Phenolics	Gaseous	1–4.8% *w*/*w*	0–10 min	[98]
Apple juice	−color −Total phenols −Chlorogenic acid −Caffeic acid −Cinnamic acid	Gaseous	0.048 mg O_3_ at a constant flow rate of 0.12 L/min	0–10 min	[76]
Banana	−Vitamin C +Total phenol +Total flavonoid	Gaseous	0.44–0.59 mmol	20 min	[85]
Black mulberry fruit (*Morus nigra*)	±color +Total soluble solids	Gaseous	2 ppm	NA	[99]
Black mulberry fruit (*M. nigra*)	+Ascorbic acid ±Total anthocyanin	Gaseous	0.64 ± 0.1 or 5.14 ± 0.1 mg ozone/m^3^	6 days	[100]
Blackberry	±Anthocyanins	Gaseous	0.1 and 0.3 ppm	12 days	[101]
Blackberry juice	−color −Anthocyanin	Gaseous	7.8% *w*/*w*	10 min	[102]
Chinese winter jujube fruit	−color +Total soluble solids +Ascorbic acid	Water	2.5 mg/L	5 min	[103]
Grape juice	−color −Anthocyanins	Gaseous	0–7.8% *w*/*w*	10 min	[104]
Guava	−Vitamin C −Total phenol −Total flavonoid	Gaseous	0.44–0.59 mmol	20 min	[98]
Guava	±Vitamin C	Water	1.1 ppm	5 min	[105]
Highbush blueberry fruit (*Vaccinum corymbosum* L.)	+Vitamin C +Flavonoids +Anthocyanins +Antioxidant activity	Gaseous	15 ppm	Intermittent 30 min, every 12 h, for 28 days	[106]
Highbush blueberry fruit (*V. corymbosum* L.)	±Antioxidant capacity ±Anthocyanins ±Phenolics	Gaseous	200 or 700 ppb	1, 2, or 4 days	[107]
Kiwifruit	+Fructose, galactose, fructose −Organic acids ±Ascorbic acid	Gaseous	2 mg/m^3^	7 months	[94]
Kiwifruit	+Soluble solids −Total phenolics ±Flavonoids +Antioxidant activity	Gaseous	300 ppb	60 days	[108]
Kiwifruit	+Total carotenoids −Total flavonoids +Total phenols +Antioxidant activity (FRAP, DPPH)	Gaseous	0.3 μL/L	for 72 or 144 h	[109]
Mandarin	±Soluble solids ±Vitamin C	Gaseous	60 mg/kg and 1.6 mg/kg	Continuous (60 mg/kg) and intermittent (1.6 mg/kg) 12 h/24 h for 28 days	[110]
Mango	+β-carotene ±Total soluble solids +Ascorbic acid ±Total phenols +Antioxidant activity (FRAP, DPPH)	Water	1 mg/L/s	20 min	[111]
Melon juice	+Phenolics +Vitamin C +Antioxidant activity	Gaseous	7.7 ± 2.4 g/L	10 min	[112]
Orange	±Soluble solids ±Vitamin C	Gaseous	60 mg/kg and 1.6 mg/kg	Continuous (60 mg/kg) and intermittent (1.6 mg/kg) 12 h/24 h for 28 days	[110]
Orange juice	−Color −Ascorbic acid	Gaseous	0.9 g/h	90 min	[113]
Orange juice	−Color −Ascorbic acid	Gaseous	0.6–10.0%	10 min	[114]
Orange juice	−Color ±Total soluble solids −Ascorbic acid −Total phenolics	Gaseous	600 mg/h	30 min	[78]
Lemon juice	−Color ±Total soluble solids −Ascorbic acid −Total phenolics	Gaseous	600 mg/h	30 min	[78]
Lime juice	−Color ±Total soluble solids −Ascorbic acid −Total phenolics	Gaseous	600 mg/h	30 min	[78]
Melon	±Color ±Carotenoids −Phenolics −Antioxidant activity	Gaseous	10.0 ± 4.8 and 38.0 ± 8.1 g/L	30 and 60 min	[115]
Papaya fruit	+Total phenolic +Antioxidant activity (FRAP, DPPH) −Ascorbic acid	Gaseous	9.2 ± 0.2 μL/L	20 and 30 min	[116]
Papaya fruit	+Total soluble solids +Ascorbic acid +β-carotene +Lycopene +Antioxidant activity	Gaseous	2.5 ppm	96 h	[117]
Peach fruit	−Color −Total phenols	Gaseous	200 mg/m^3^	Intermittent 30 min every week for 60 days	[118]
Pear	+Total phenolics +Total flavonoids	Gaseous	6.42 mg/m^3^	Intermittent 1 h/d for 8 days	[119]
Persimmon	±Color index ±Total soluble solids	Gaseous	0.15 ppm (*vol*/*vol*)	30 days	[120]
Pineapple	−Vitamin C +Total phenol +Total flavonoid	Gaseous	0.44–0.59 mmol	20 min	[98]
Pitaya juice	−Color −Total betalain −Betaxanthins −Betacyanins −Total phenolics −Antioxidant activity (DPPH, ABTS)	Gaseous	24 mg/L/min	7 min	[88]
Pummelo juice	−Total phenolics −Ascorbic acid	Gaseous	600 mg/h	50 min	[121]
Raspberry (*Rubus ideaeus* L.)	±Color +Soluble solids +Phenols +Flavonoids +Anthocyanins +Vitamin C +Total antioxidant activity	Gaseous	0.3 and 0.9 mg/L	60 and 120 min	[40]
Raspberry (*Rubus ideaeus* L.)	+Antioxidant capacity +Phenolics +Anthocyanins ±Vitamin C	Gaseous	8–10 ppm	Intermittent 30 min, every 12 h, for 3 days	[39]
Raspberry	±Soluble solids −Color ±Anthocyanins ±Phenols ±Antioxidant capacity	Gaseous	50 and 200 ppb	12 h	[122]
Mandarin	+Flavonoid content +Antioxidant activity (DPPH, ABTS)	Gaseous	2.5 μg/L	24 h	[123]
Strawberry	+Total phenols +Total flavonoids +Total anthocyanins	Gaseous	5 ppm	Intermittent 10 h every week for 21 days	[124]
Strawberry	+Color +Phenolics +Anthocyanin	Water	0.1 ppm	2 min	[125]
Strawberry	−Anthocyanin +Vitamin C −Sucrose, glucose, fructose −Volatile/aroma compounds	Gaseous	0.35 ppm	3 days	[126]
Strawberry	−Phenolics −Procyanidins −Vitamin C	Gaseous	5, 10, 15, and 20 g/L	NA	[127]
Strawberry	+Ascorbic acid	Gaseous	4 ppm	Intermittent 30 min every day for 20 days	[128]
Strawberry	+Phenolics +Antioxidant capacity	Gaseous	0.3–1.2 mg/L	60, 120, 150, and 180 min	[129]
Strawberry juice	−Anthocyanin −Ascorbic acid −color	Gaseous	7.8% *w*/*w*	10 min	[130]
Table grape berries	+Phytoalexins (resveratrol, pterostilbene)	Gaseous	16 mg/L	5–10 min	[131]
Table grapes	+Total flavanols +Total amount of hydroxycinnamic acid derivatives +Total phenolics	Gaseous	0.1 µL/L	60 days	[90]
Table grapes (white)	+Stilbenoids −Color	Gaseous	1.67 and 3.88 g/h	1, 3, and 5 h	[132]
Wine grapes (white)	+Catechin −Hydroxycinnamic acids −Antiradical capacity ±Total soluble solid −Total polyphenols −Flavanols	Gaseous	1.5 g/h	12 h	[93]
Wine red grapes	+Polyphenols ±Carotenoids ±Anthocyanin	Gaseous	1.5 g/h	18 h	[23]
Wine red grapes	−Polyphenols −Carotenoids −Anthocyanin	Gaseous	0.5 g/h and 1.5 g/h	Continuous 18 h and intermittent 4 h/day	[23]
Wine grapes	±Total soluble solids +Polyphenols	Gaseous	20 g/h with 6% *w*/*w*	12 h	[133]
Adzuki beans (*Vigna angularis*)	±Total phenolics ±Antioxidant capacity (DPPH, ABTS)	Gaseous	62 mg/L	120 min	[134]
Bell pepper (*Capsicum annuum* L.)	−Color −Chlorophyll −Ascorbic acid ±Soluble solid	Gaseous	7 and 9 ppm	3 days	[135]
Bell pepper (*C. annuum* L.)	±Color −Chlorophyll +Ascorbic acid ±Soluble solid	Gaseous	1 and 3 ppm	3 days	[135]
Bell pepper (shredded green)	+Ascorbic acid +Color	Water	1–3 mg/L	1–5 min	[136]
Bell pepper (frozen green)	±Color ±Chlorophyll −Dehydroascorbic acid −Free ascorbic acid −Total ascorbic acid	Gaseous	10 ppm	5 min	[137]
Broccoli (*Brassica oleracea* L.)	±Polyphenolic ±Flavonoids ±Vitamin C ±Total chlorophyll	Water	NA	5 and 10 min	[138]
Carrots	−Ascorbic acid, −Carotenoids	Water	1:2 *w*/*v*; 200 mg O_3_/h	10 min	[139]
Carrots	±Glucose, fructose, and galactose	Gaseous	50 ± 10 nL/L	NA	[140]
Carrots	±Color +Soluble solids	Gaseous and water	0–10 mg/L	30,60, and 90 min	[141]
Carrots (shredded)	−Total phenols −Antioxidant capacity −Soluble solids	Water	2 ppm	60 min	[79]
Celery (fresh-cut)	±Vitamin C ±Total sugar	Water	0.03, 0.08, and 0.18 ppm	5 min	[142]
Chicory	+Total antioxidant activity ±Sesquiterpene lactones +Chicoric acid ±Sugars	Gaseous	8.25 mg/L (from first day) and 9 mg/L (from third day till end)	NA	[91]
Chili peppers	±Color ±Lycopene +Fructose +Ascorbic acid ±Total phenolics ±Antioxidant activity	Gaseous	0.45 and 0.9 μmol/mol	NA	[143]
Chili peppers	−Color −Glucose +Ascorbic acid ±Total phenolics −Antioxidant activity	Gaseous	2 μmol/mol	NA	[143]
Chili peppers	+Ascorbic acid +Antioxidant activity ±Capsaicin ±Total phenolics ±Total flavonoids −Total carotenoids +Total chlorophyll	Gaseous	30 mg/L	10 min	[144]
Green asparagus	+Antioxidant activity	Water	1 mg/L	30 min	[145]
Hot red pepper fruits	+Quercetin 3-*O*-rhamnoside +Quercetin 3-*O*-rhamnoside-7-*O*-glucoside +total phenolics +Antiradical activity (DPPH)	Gaseous	2 mg/L	1 h and 3 h	[86]
Hot red pepper fruits	+Phenolics +Antioxidant activity	Gaseous	2 mg/L	3 h	[41]
Lettuce	±Chlorophyll ±Ascorbic acid ±Total phenolics ±Antioxidant activity	Water	12 mg/L	15 min	[61]
Lettuce (green leaf) (*Lactuca sativa*)	±Vitamin C ±β-carotene	Water	2 ppm	2 min	[146]
Lettuce (iceberg)	±color ±β-carotene ±Vitamin C	Water	4 mg/L	15 min	[147]
Lettuce (iceberg)	±Phenolics −Ascorbic acid	Water	10 and 20 mg/L	3 min	[148]
Lettuce (iceberg)	±Total organic carbon ±Vitamin C +Sugar content	Water	3.6 ppm	NA	[149]
Parsley leaves	±Chlorophyll −Ascorbic acid −Total phenolics −Antioxidant activity	Gaseous	950 ± 12 µL/L	20 min	[61]
Rocket (*Eruca sativa* Mill.) leaves	±Phenolics ±Total antioxidant capacity ±Total chlorophyll ±Carotenoids ±Ascorbic acid	Gaseous	1, 5, and 10 ppm	10 min	[150]
Spinach	±Chlorophyll ±Ascorbic acid ±Total phenolics ±Antioxidant activity	Water	12 mg/L	15 min	[61]
Tomato	±Soluble sugars (glucose, fructose) ±Antioxidant status ±Vitamin C ±Total phenolics +β-carotene +Lutein +Lycopene	Gaseous	1.0 μmol/mol	1 and 6 days	[59]
Tomato	+Vitamin C	Gaseous	1 ppm/vol	24 h	[151]
Tomato	+Total antioxidant activity ±Color	Gaseous	0.9 and 2.5 mg/L	30 and 120 min	[152]
Tomato	+Fructose, glucose +Ascorbic acid +Fumaric acid	Gaseous	4 ± 0.5 μL/L	Intermittent for 30 min every 3 h for 15 days	[92]
Tomato	±Soluble solids −Lycopene ±Vitamin C	Gaseous	25 mg/m^3^ and 45 mg/m^3^	Intermittent 2 h/d for 16 days	[153]
Tomato (grape)	−Phenolic compounds −Lycopene −Vitamin C	Gaseous	3.43 mg/L and 6.85 mg/L	2 and 4 h	[154]
Tomato (*Solanum lycopersicum* L.)	±Color ±Sugar content +Phenolic compounds ±Antioxidant capacity	Gaseous	10 μL/L	10 min	[84]
Tomato juice	−color −Ascorbic acid	Gaseous	7.8% *w*/*w*	10 min	[155]

NA—Not available.

## References

[1] Weng C.J., Yen G.C. (2012). Chemopreventive effects of dietary phytochemicals against cancer invasion and metastasis: Phenolic acids, monophenol, polyphenol, and their derivatives. Cancer Treat. Rev..

[2] Haminiuk C.W.I., Maciel G.M., Plata-Oviedo M.S.V., Peralta R.M. (2012). Phenolic compounds in fruits—An overview. Int. J. Food Sci..

[3] Ahn-Jarvis J.H., Parihar A., Dose A.I. (2019). Dietary flavonoids for immunoregulation and cancer: Food Design for Targeting Disease. Antioxidants.

[4] Flis S., Jastrzębski Z., Namiesnik J., Arancibia-Avila P., Toledo F., Leontowicz H., Leontowicz M., Suhaj M., Trakhtenberg S., Gorinstein S. (2012). Evaluation of inhibition of cancer cell proliferation in vitro with different berries and correlation with their antioxidant levels by advanced analytical methods. J. Pharm. Biomed. Anal..

[5] Caillet S., Lorenzo G., Côté J., Doyon G., Sylvain J.F., Lacroix M. (2012). Cancer chemopreventive effect of fractions from cranberry products. Food Res. Int..

[6] Noratto G., Porter W., Byrne D., Cisneros-Zevallos L. (2009). Identifying peach and plum polyphenols with chemopreventive potential against estrogen-independent breast cancer cells. J. Agric. Food Chem..

[7] Aiyer H.S., Warri A.M., Woode D.R., Hilakivi-Clarke L., Clarke R. (2012). Influence of berry polyphenols on receptor signalling and cell-death pathways: Implications for breast cancer prevention. J. Agric. Food Chem..

[8] Kumar A., Butt N.A., Levenson A.S., Tollefsbol T.O. (2016). Natural epigenetic-modifying molecules in medical therapy. Medical Epigenetics.

[9] Brown E.M., Gill C.I.R., McDougall G.J., Stewart D. (2012). Mechanisms underlying the anti-proliferative effects of berry components in in vitro models of colon cancer. Curr. Pharm. Biotechnol..

[10] Ferysiuk K., Wójciak K.M., Materska M., Chilczuk B., Pabich M. (2020). Modification of lipid oxidation and antioxidant capacity in canned refrigerated pork with a nitrite content reduced by half and addition of sweet pepper extract. LWT.

[11] Ning J., Hou G.G., Sun J., Wan X., Dubat A. (2017). Effect of green tea powder on the quality attributes and antioxidant activity of whole-wheat flour pan bread. LWT.

[12] Dang Vu K., Carlettini H., Bouvet J., Côté J., Doyon G., Sylvain J.F., Lacroix M. (2012). Effect of different cranberry extracts and juices during cranberry juice processing on the antiproliferative activity against two colon cancer cell lines. Food Chem..

[13] Roohinejad S. (2018). Application of plant extracts to improve the shelf-life, nutritional andhealth-related properties of ready-to-eat meat products. Meat Sci..

[14] Kambiranda D.M., Basha S.M., Stringer S.J., Obuya J.O., Snowden J.J. (2019). Multi-year quantitative evaluation of stilbenoids levels among selected muscadine grape cultivars. Molecules.

[15] Żebrowska J., Dyduch-Siemińska M., Gawroński J., Jackowska I., Pabich M. (2019). Genetic estimates of antioxidant properties in the conventionally and *in vitro* propagated strawberry (*Fragaria* × *ananassa* Duch.). Food Chem..

[16] Ghimire B.K., Yu C.Y., Ghimire B., Seong E.S., Chung I.M. (2019). Allelopathic potential of phenolic compounds in *Secale cereale* cultivars and its relationship with seeding density. Appl. Sci..

[17] Fang L., Yang T., Medina-Bolivar F. (2020). Production of prenylated stilbenoids in hairy root cultures of peanut (*Arachis hypogaea*) and its wild relatives *A. ipaensis* and *A. duranensis* via an optimized elicitation procedure. Molecules.

[18] Moreira M.R., Cassani L., Martín-Belloso O., Soliva-Fortuny R. (2015). Effects of polysaccharide-based edible coatings enriched with dietary fiber on quality attributes of fresh-cut apples. J. Food Sci. Technol..

[19] Ziarno M., Zaręba D. (2015). The Use of ozone to destroy microorganisms. Przem. Chem..

[20] Selma M.V., Ibáñez A.M., Cantwell M., Suslow T. (2008). Reduction by gaseous ozone of *Salmonella* and microbial flora associated with fresh-cut cantaloupe. Food Microbiol..

[21] Kim J.G., Yousef A.E., Dave S. (1999). Application of Ozone for enhancing the microbiological safety and quality of foods: A review. J. Food Prot..

[22] Pankaj S.K., Shi H., Keener K.M. (2018). A review of novel physical and chemical decontamination technologies for aflatoxin in food. Trends Food Sci. Technol..

[23] Botondi R., De Sanctis F., Moscatelli N., Vettraino A.M., Catelli C., Mencarelli F. (2015). Ozone fumigation for safety and quality of wine grapes in postharvest dehydration. Food Chem..

[24] Sung H.J., Song W.J., Kim K.P., Ryu S., Kang D.H. (2014). Combination effect of ozone and heat treatments for the inactivation of *Escherichia coli* O157:H7, *Salmonella Typhimurium*, and *Listeria monocytogenes* in apple juice. Int. J. Food Microbiol..

[25] Alwi N.A., Ali A. (2014). Reduction of *Escherichia coli* O157, *Listeria monocytogenes* and *Salmonella enterica* sv. Typhimurium populations on fresh-cut bell pepper using gaseous ozone. Food Control.

[26] Ding W., Jin W., Cao S., Zhou X., Wang J.Q., Huang H., Tu R., Han S.H., Wang Q. (2019). Ozone disinfection of chlorine-resistant bacteria in drinking water. Water Res..

[27] Khadre M.S., Yousef A.E., Kim J.-G. (2001). Microbiological aspects of ozone applications in food: A review. J. Food Sci..

[28] Minas I.S., Tanou G., Krokida A., Karagiannis E., Belghazi M., Vasilakakis M., Papadopoulou K.K., Molassiotis A. (2018). Ozone-induced inhibition of kiwifruit ripening is amplified by 1-methylcyclopropene and reversed by exogenous ethylene. BMC Plant Biol..

[29] De Souza L.P., Faroni L.R.D., Heleno F.F. (2017). Ozone treatment for pesticide removal from carrots: Optimization by response surface methodology. Food Chem..

[30] Sadło S., Szpyrka E., Piechowicz B., Antos P., Balawejder M. (2017). Reduction of captan, boscalid and pyraclostrobin residues on apples using water only, gaseous ozone and ozone aqueous solution. Ozone Sci. Eng..

[31] Heleno F.F., de Queiroz M.E., Neves A.A., Freitas R.S., Faroni L.R., de Oliveira A.F. (2014). Effects of ozone fumigation treatment on the removal of residual difenoconazole from strawberries and on their quality. J. Environ. Sci. Health Part B.

[32] Wang L., Luo Y., Luo X., Wang R., Li Y., Li Y., Shao H., Chen Z. (2016). Effect of deoxynivalenol detoxification by ozone treatment in wheat grains. Food Control.

[33] Pascari X., Ramos A.J., Marín S., Sanchís V. (2018). Mycotoxins and beer. Impact of beer production process on mycotoxin contamination. A review. Food Res. Int..

[34] Trombete F., Porto Y., Freitas-Silva O., Pereira R., Direito G., Saldanha T., Fraga M. (2017). Efficacy of ozone treatment on mycotoxins and fungal reduction in artificially contaminated soft wheat grains. J. Food Process. Preserv..

[35] Zhu F. (2018). Effect of ozone treatment on the quality of grain products. Food Chem..

[36] Luo X., Wang R., Wang L., Li Y., Bian Y., Chen Z. (2014). Effect of ozone treatment on aflatoxin B1 and safety evaluation of ozonized corn. Food Control.

[37] Pandiselvam R., Kaavya R., Yasendra J., Kornautchaya V., Piraya L., Divya V., Anjineyulu K., Ramesh S.V. (2020). Ozone as a novel emerging technology for the dissipation of pesticide residues in foods–a review. Trends Food Sci. Technol..

[38] Velioglu S., Ergen Ş.F., Aksu P., Altındağ A. (2018). Effects of ozone treatment on the degradation and toxicity of several pesticides in different groups. J. Agric. Sci..

[39] Piechowiak T., Antos P., Kosowski P., Skrobacz K., Józefczyk R., Balawejder M. (2019). Impact of ozonation process on the microbiological and antioxidant status of raspberry (*Rubus ideaeus* L.) fruit during storage at room temperature. Agr. Food Sci..

[40] Onopiuk A., Półtorak A., Moczkowska M., Szpicer A., Wierzbicka A. (2017). The impact of ozone on health-promoting, microbiological, and colour properties of *Rubus ideaus* raspberries. CyTA J. Food.

[41] Sachadyn-Król M., Materska M., Chilczuk B. (2019). Ozonation of hot red pepper fruits increases their antioxidant activity and changes some antioxidant contents. Antioxidants.

[42] Martínez-Sánchez A., Aguayo E. (2020). Effects of ozonated water irrigation on the quality of grafted watermelon seedlings. Sci. Hortic..

[43] Ahmad E., Arshad M., Khan M.Z., Amjad M.S., Sadaf H.M., Riaz I., Sabir S., Sabaoon N.A. (2017). Secondary metabolites and their multidimensional prospective in plant life. J. Pharmacogn. Phytochem..

[44] Gawlik-Dziki U., Dziki D., Nowak R., Świeca M., Olech M., Pietrzak W. (2016). Influence of sprouting and elicitation on phenolic acids profile and antioxidant activity of wheat seedlings. J. Cereal Sci..

[45] Baenas N., Garcıa-Viguera C., Moreno D.A. (2014). Elicitation: A tool for enriching the bioactive composition of foods. Molecules.

[46] Thakur M., Bhattacharya S., Khosla P.K., Puri S. (2019). Improving production of plant secondary metabolites through biotic and abiotic elicitation. J. Appl. Res. Med. Aromat. Plants.

[47] Złotek U., Świeca M., Jakubczyk A. (2014). Effect of abiotic elicitation on main health-promoting compounds, antioxidant activity and commercial quality of butter lettuce (*Lactuca sativa* L.). Food Chem..

[48] Viacava G.E., Goyeneche R., Goñi M.G., Roura S.I., Agüero M.V. (2018). Natural elicitors as preharvest treatments to improve postharvest quality of Butterhead lettuce. Sci. Hortic..

[49] Świeca M., Baraniak B. (2014). Nutritional and antioxidant potential of lentil sprouts affected by elicitation with temperature stress. J. Agric. Food Chem..

[50] Natella F., Maldini M., Nardini M., Azzini E., Foddai M.S., Giusti A.M., Baima S., Morelli G., Scaccini C. (2016). Improvement of the nutraceutical quality of broccoli sprouts by elicitation. Food Chem..

[51] Złotek U. (2017). Effect of jasmonic acid and yeast extract elicitation on low-molecular antioxidants and antioxidant activity of marjoram (*Origanum majorana* L.). Acta Sci. Pol. Technol. Aliment..

[52] Puthusseri B., Divya P., Lokesh V., Neelwarne B. (2012). Enhancement of folate content and its stability using food grade elicitors in coriander (*Coriandrum sativum* L.). Plant Foods Hum. Nutr..

[53] Flores G., Ruiz Del Castillo M.L. (2016). Accumulation of anthocyanins and flavonols in black currants (*Ribes nigrum* L.) by pre-harvest methyl jasmonate treatments. J. Sci. Food Agric..

[54] Kumari R.S.B., Agrawal S., Singh N.K.D. (2009). Supplemental ultraviolet-B induced changes in essential oil composition and total phenolics of *Acorus calamus* L. (sweet flag). Ecotoxicol. Environ. Saf..

[55] Parida A.K., Das A.B. (2005). Salt tolerance and salinity effects on plants: A review. Ecotoxicol. Environ. Saf..

[56] Rudolf J.R., Resurreccion A.V. (2005). Elicitation of resveratrol in peanut kernels by application of abiotic stresses. J. Agric. Food Chem..

[57] Darré M., Valerga L., Araque L.C.O., Lemoine M.L., Demkura P.V., Vicente A.R. (2017). Role of UV-B irradiation dose and intensity on color retention and antioxidant elicitation in broccoli florets (*Brassica oleracea*, var. italica). Postharvest Biol. Technol..

[58] De la Peña Moreno F., Monagas M., Blanch G.P., Bartolomé B., Ruiz del Castillo M.L. (2010). Enhancement of anthocyanins and selected aroma compounds in strawberry fruits through methyl jasmonate vapor treatment. Eur. Food Res. Technol..

[59] Tzortzakis N., Borland A., Singleton I., Barnes J. (2007). Impact of atmospheric ozone-enrichment on quality-related attributes of tomato fruit. Postharvest Biol. Technol..

[60] Wu Q., Zhang Z., Zhu H., Li T., Zhu X., Gao H., Yun Z., Jiang Y. (2019). Comparative volatile compounds and primary metabolites profiling of pitaya fruit peel after ozone treatment. J. Sci. Food Agric..

[61] Karaca H., Velioglu S. (2014). Effects of ozone treatments on microbial quality and some chemical properties of lettuce, spinach, and parsley. Postharvest Biol. Technol..

[62] Oksanen E., Häikiö E., Sober J., Karnosky D. (2004). Ozone-induced H_2_O_2_ accumulation in field-grown aspen and birch is linked to foliar ultrastructure and peroxisomal activity. New Phytol..

[63] Yaseen T., Ricelli A., Turan B., Albanese P., D’onghia A.M. (2015). Ozone for post-harvest treatment of apple fruits. Phytopathol. Mediterr..

[64] Damara E. (2017). Ozone and Electrolyzed Water Application to Preserve Quality of Citrus Fruit and Effect of Gene Expression Related to Plant Defense Mechanisms. Ph.D. Thesis.

[65] Ludwikow A., Sadowski J. (2008). Gene networks in plant ozone stress response and tolerance. J. Integr. Plant Biol..

[66] Campayo A., Serrano de la Hoz K., García-Martínez M.M., Sánchez-Martínez J.F., Salinas M.R., Alonso G.L. (2019). Spraying ozonated water on Bobal grapevines: Effect on grape quality. Food Res. Int..

[67] Flores P., Hernández V., Fenoll J., Hellín P. (2019). Pre-harvest application of ozonated water on broccoli crops: Effect on head quality. J. Food Compos. Anal..

[68] Martínez-Sánchez A., Encarna A. (2019). Effect of irrigation with ozonated water on the quality of capsicum seedlings grown in the nursery. Agric. Water Manag..

[69] Fernandes F.F., Esposito M.p., da Silva Engela M.R.G., Cardoso-Gustavson P., Furlan C.M., Hoshika Y., Carrari E., Magni G., Domingos M., Paoletti E. (2019). The passion fruit liana (*Passiflora edulis* Sims, Passifloraceae) is tolerant to ozone. Sci. Total Environ..

[70] Bortolin R.C., Caregnato F.F., Divan Junior A.M., Zanotto-Filho A., Moresco K.S., Rios A.D.O., Salvi Ade O., Ortmann C.F., de Carvalho P., Reginatto F.H. (2016). Chronic ozone exposure alters the secondary metabolite profile, antioxidant potential, anti-inflammatory property, and quality of red pepper fruit from *Capsicum baccatum*. Ecotoxicol. Environ. Saf..

[71] Keutgen A.J., Pawelzik E. (2008). Influence of preharvest ozone exposure on quality of strawberry fruit under simulated retail conditions. Postharvest Biol. Technol..

[72] Horvitz S., Cantalejo M.J. (2014). Application of ozone for the postharvest treatment of fruits and vegetables. Crit. Rev. Food Sci. Nutr..

[73] Segade S.R., Vincenzi S., Giacosa S., Rolle L. (2019). Changes in stilbene composition during postharvest ozone treatment of ‘Moscato bianco’ winegrapes. Food Res. Int..

[74] Tomás-Barberán F.A., Espín J.C. (2001). Phenolic compounds and related enzymes as determinants of quality in fruits and vegetables. J. Sci. Food Agric..

[75] Cullen P.J., Tiwari B.K., O’Donnell C.P., Muthukumarappan K. (2009). Modelling approaches to ozone processing of liquid foods. Trends Food Sci. Technol..

[76] Patil S., Torres B., Tiwari B.K., Hilde Wijngaard H., Bourke P., Cullen P.J., O’ Donnell C.P., Valdramidis V.P. (2010). Safety and quality assessment during the ozonation of cloudy apple juice. J. Food Sci..

[77] Criegee R. (1975). Mechanism of ozonolysis. Angew. Chem. Int. Ed. Engl..

[78] Shah N.N.A.K., Sulaiman A., Sidek N.S.M., Supian N.A.M. (2019). Quality assessment of ozone-treated citrus fruit juices. Int. Food Res. J..

[79] Augspole I., Rakcejeva T., Dukalska L., Skudra L. (2014). Providing quality of shredded carrots during storage by treatment with ozonated water. Mater. Sci. Appl. Chem..

[80] Zou Y., Yang M., Zhang G., He H., Yang T. (2015). Antioxidant activities and phenolic compositions of wheat germ as affected by the roasting process. J. Am. Oil Chem. Soc..

[81] Paissoni M.A., Segade S.R., Giacosa S., Torchio F., Cravero F., Englezos V., Rantsiou K., Carboni C., Gerbi V., Teissedre P.L. (2017). Impact of post-harvest ozone treatments on the skin phenolic extractability of red winegrapes cv Barbera and Nebbiolo (*Vitis vinifera* L.). Food Res. Int..

[82] Ranieri A., D’Urso G., Nali C., Lorenzini G., Soldatini G.F. (1996). Ozone stimulates apoplastic antioxidant systems in pumpkin leaves. Physiol. Plant..

[83] Wiese C.B., Pell E.J. (2003). Oxidative modification of the cell wall in tomato plants exposed to ozone. Plant Physiol. Biochem..

[84] Rodoni L., Casadei N., Concellón A., Chaves Alicia A.R., Vicente A.R. (2010). Effect of short-term ozone treatments on tomato (*Solanum lycopersicum* L.) fruit quality and cell wall degradation. J. Agric. Food Chem..

[85] Alothman M., Kaur B., Fazilah A., Bhat R., Karim A.A. (2010). Ozone-induced changes of antioxidant capacity of fresh-cut tropical fruits. Innov. Food Sci. Emerg. Technol..

[86] Sachadyn-Król M., Materska M., Chilczuk B., Karaś M., Jakubczyk A., Perucka I., Jackowska I. (2016). Ozone-induced changes in the content of bioactive compounds and enzyme activity during storage of pepper fruits. Food Chem..

[87] de Souza Sartori J.A., Angolini C.F., Eberlin M.N., Aguiar C.L. (2017). Criegee mechanism as a safe pathway of color reduction in sugarcane juice by ozonation. Food Chem..

[88] Asokapandian S., Periasamy S., Swamy G., Rajauria G., Tiwari B.K. (2018). Ozone for fruit juice preservation. Fruit Juices: Extraction, Composition, Quality and Analysis.

[89] García-Mateos M.R., Quiroz-González B., Corrales-García J., Ybarra-Moncada C., Leyva-Ruelas G. (2019). Ozone-high hydrostatic pressure synergy for the stabilization of refrigerated pitaya (*Stenocereus pruinosus*) juice. Innov. Food Sci. Emerg. Technol..

[90] Artés-Hernández F., Aguayo E., Artés F., Tomás-Barberán F. (2007). Enriched ozone atmosphere enhances bioactive phenolics in seedless table grapes after prolonged shelf life. J. Sci. Food Agric..

[91] Nicoletto C., Maucieri C., Sambo P. (2017). Effects on water management and quality characteristics of ozone application in chicory forcing process: A pilot system. Agronomy.

[92] Aguayo E., Escalona V.H., Artes F. (2006). Effect of cyclic exposure to ozone gas on physicochemical, sensorial and microbial quality of whole and sliced tomatoes. Postharvest Biol. Tec..

[93] Carbone K., Mencarelli F. (2015). Influence of short-term postharvest ozone treatments in nitrogen or air atmosphere on the metabolic response of white wine grapes. Food Bioproc. Technol..

[94] Barboni T., Cannac M., Chiaramonti N. (2010). Effect of cold storage and ozone treatment on physicochemical parameters, soluble sugars and organic acids in *Actinidia deliciosa*. Food Chem..

[95] Lv Y., Tahir I., Olsson M.E. (2019). Effect of ozone application on bioactive compounds of apple fruit during short-term cold storage. Sci. Hortic..

[96] Swami S., Muzammil R., Saha S., Shabeer A., Oulkar D., Banerjee K., Singh S.B. (2016). Evaluation of ozonation technique for pesticide residue removal and its effect on ascorbic acid, cyanidin-3-glucoside, and polyphenols in apple (*Malus domesticus*) fruits. Environ. Monit. Assess..

[97] Liu C., Ma T., Hu W., Tian M., Sun L. (2016). Effects of aqueous ozone treatments on microbial load reduction and shelf life extension of fresh-cut apple. Int. J. Food Sci. Technol..

[98] Torres B., Tiwari B.K., Patras A., Wijngaard H.H., Brunton N., Cullen P.J., O’Donnell C.P. (2011). Effect of ozone processing on the colour, rheological properties and phenolic content of apple juice. Food Chem..

[99] Han Q., Gao H.Y., Chen H.J., Fang X.J., Wu W.J. (2017). Precooling and ozone treatments affects postharvest quality of black mulberry (*Morus nigra*) fruits. Food Chem..

[100] Tabakoglu N., Karaca H. (2018). Effects of ozone-enriched storage atmosphere on postharvest quality of black mulberry fruits (*Morus nigra* L.). LWT Food Sci. Technol..

[101] Barth M.M., Zhou C., Mercier J., Payne F.A. (1995). Ozone storage effects on anthocyanin content and fungal growth in blackberries. J. Food Sci..

[102] Tiwari B.K., O’donnell C.P., Muthukumarappan K., Cullen P.J. (2009). Anthocyanin and colour degradation in ozone treated blackberry juice. Innov. Food Sci. Emerg. Technol..

[103] Li H., Xiong Z., Gui D., Li X. (2019). Effect of aqueous ozone on quality and shelf life of Chinese winter jujube. J. Food Process. Preserv..

[104] Tiwari B.K., O’Donnell C.P., Patras A., Brunton N., Cullen P.J. (2009). Anthocyanins and color degradation in ozonated grape juice. Food Chem. Toxicol..

[105] Setiasih I.S., Rialita T., Sumanti D.M., Hanidah I. (2017). Characteristics of guava (*Psidium guajava* L.) treated with ozonation during ambient storage. KnE Life Sci..

[106] Piechowiak T., Antos P., Józefczyk R., Kosowski P., Skrobacz K., Balawejder M. (2018). Impact of ozonation process on the microbiological contamination and antioxidant capacity of highbush blueberry (*Vaccinum corymbosum,* L.) fruit during cold storage. Ozone Sci. Eng..

[107] Song J., Fan L., Forney C.F., Jordan M.A., Hildebrand P.D., Kalt W., Ryan D.A.J. (2003). Effect of ozone treatment and controlled atmosphere storage on quality and phytochemicals in highbush blueberries. Acta Hortic..

[108] Goffi V., Zampella L., Forniti R., Petriccione M., Botondi R. (2019). Effects of ozone postharvest treatment on physicochemical and qualitative traits of *Actinidia chinensis* ‘Soreli’ during cold storage. J. Sci. Food Agric..

[109] Minas I.S., Karaoglanidis G.S., Manganaris G.A., Vasilakakis M. (2012). Effect of ozone application during cold storage of kiwifruit on the development of stem-end rot caused by *Botrytis cinerea*. Postharvest Biol. Technol..

[110] De Almeida Monaco K., Costa S.M., Minatel I.O., Correa C.R., Calero F.A., Vianello F., Lima G.P.P. (2016). Influence of ozonated water sanitation on postharvest quality of conventionally and organically cultivated mangoes after postharvest storage. Postharvest Biol. Technol..

[111] García-Martín J.F., Olmo M., García J.M. (2018). Effect of ozone treatment on postharvest disease and quality of different citrus varieties at laboratory and at industrial facility. Postharvest Biol. Technol..

[112] Sroy S., Fundo J.F., Miller F.A., Brandão T.R.S., Silva C.L.M. (2019). Impact of ozone processing on microbiological, physicochemical, and bioactive characteristics of refrigerated stored *Cantaloupe* melon juice. J. Food Process. Preserv..

[113] Angelino P.D., Golden A., Mount J.R. (2003). Effect of ozone treatment on quality of orange juice. IFT Annual Meeting Book of Abstracts, Chicago, IL, USA, 12–16 July 2003.

[114] Tiwari B.K., Muthukumarappan K., O’Donnell C.P., Cullen P.J. (2008). Kinetics of freshly squeezed orange juice quality changes during ozone processing. J. Agric. Food Chem..

[115] Miller F.A., Fundo J.F., Silva C.L.M., Brandão T.R.S. (2018). Physicochemical and bioactive compounds of ‘Cantaloupe’ melon: Effect of ozone processing on pulp and seeds. Ozone Sci. Eng..

[116] Yeoh W.K., Ali A., Forney C.F. (2014). Effects of ozone on major antioxidants and microbial populations of fresh-cut papaya. Postharvest Biol. Technol..

[117] Ali A., Ong M.K., Forney C.F. (2014). Effect of ozone pre-conditioning on quality and antioxidant capacity of papaya fruit during ambient storage. Food Chem..

[118] Jia X., Li J., Du M., Zhao Z., Song J., Yang W., Zheng Y., Chen L., Li X. (2020). Combination of low fluctuation of temperature with TiO_2_ photocatalytic/ozone for the quality maintenance of postharvest peach. Foods.

[119] Zhao Z., Xu G., Han Z., Li Q., Chen Y., Li D. (2013). O_3_ Affects antioxidant capacity of pear. J. Food Qual..

[120] Salvador A., Abad I., Arnal L., Martínez-Jávega J. (2006). Effect of ozone on postharvest quality of persimmon. J. Food Sci..

[121] Shah N.N.A.K., Supian N.A.M., Hussein N.A. (2019). Disinfectant of pummelo (*Citrus Grandis* L. Osbeck) fruit juice using gaseous ozone. J. Food Sci. Technol..

[122] Giuggioli N., Briano R., Girgenti V., Peano C. (2015). Quality effect of ozone treatment for the red raspberries storage. Chem. Eng. Trans..

[123] Zhu X., Jiang J., Yin C., Li G., Jiang Y., Shan Y. (2019). Effect of ozone treatment on flavonoid accumulation of satsuma mandarin (*Citrus unshiu* Marc.) during ambient storage. Biomolecules.

[124] Chen C.K., Zhang H.J., Dong C.H., Ji H.P., Zhang X.J., Li L., Ban Z.J., Zhang N., Xue W.T. (2019). Effect of ozone treatment on the phenylpropanoid biosynthesis of postharvest strawberries. RSC Adv..

[125] Nayak S.L., Sethi S., Sharma R.R., Sharma R.M., Singh S., Singh D. (2020). Aqueous ozone controls decay and maintains quality attributes of strawberry (*Fragaria ananassa* Duch.). J. Food Sci. Technol..

[126] Pérez A.G., Sanz C., Ríos J.J., Olías R., Olías J.M. (1999). Effects of ozone treatment on postharvest strawberry quality. J. Agric. Food Chem..

[127] Allende A., Marín A., Buendía B., Tomás-Barberán F., Gil M.I. (2007). Impact of combined postharvest treatments (UV-C light, gaseous O_3_, superatmospheric O_2_ and high CO_2_) on health promoting compounds and shelf-life of strawberries. Postharvest Biol. Tec..

[128] Zhang X., Zhang Z., Wang L., Zhang Z., Li J., Zhao C. (2011). Impact of ozone on quality of strawberry during cold storage. Front. Agric. China.

[129] Onopiuk A., Półtorak A., Wyrwisz J., Moczkowska M., Stelmasiak A., Lipińska A., Arkadiusz Szpicer A., Zalewska M., Zaremba R., Kuboń M. (2017). Impact of ozonisation on pro-health properties and antioxidant capacity of ‘Honeoye’ strawberry fruit. CyTA-J. Food.

[130] Tiwari B.K., O’Donnell C.P., Patras A., Brunton N., Cullen P.J. (2009). Effect of ozone processing on anthocyanins and ascorbic acid degradation of strawberry juice. Food Chem..

[131] Sarig P., Zahavi T., Zutkhi Y., Yannai S., Lisker N., Ben-Arie R. (1996). Ozone for control of post-harvest decay of table grapes caused by *Rhizopus stolonifera*. Physiol. Mol. Plant Pathol..

[132] González-Barrio R., Beltrán D., Cantos E., Gil M.I., Espín J.C., Tomás-Barberán F.A. (2006). Comparison of ozone and UV-C treatments on the postharvest stilbenoid monomer, dimer, and trimer induction in var. ‘Superior’ white table grapes. J. Agric. Food Chem..

[133] Modesti M., Petriccione M., Forniti R., Zampella L., Scortichini M., Mencarelli F. (2018). Methyl jasmonate and ozone affect the antioxidant system and the quality of wine grape during postharvest partial dehydration. Food Res. Int..

[134] Alexandre A.P.S., Miano A.C., Brandão T.R.S., Miller F.A., Fundo J.F., Calori-Domingues M.A., Silva C.L.M., Augusto P.E.D. (2018). Ozonation of Adzuki beans (*Vigna angularis*): Effect on the hydration kinetics, phenolic compounds and antioxidant capacity. J. Food Process Eng..

[135] Alwi N.A., Ali A. (2015). Dose-dependent effect of ozone fumigation on physiological characteristics, ascorbic acid content and disease development on bell pepper (*Capsicum annuum* L.) during storage. Food Bioproc. Technol..

[136] Ummat V., Singh A.K., Sidhu G.K. (2018). Effect of aqueous ozone on quality and shelf life of shredded green bell pepper (*Capsicum annuum*). J. Food Process. Preserv..

[137] Maherani B., Harich M., Salmieri S., Lacroix M. (2019). Antibacterial properties of combined non-thermal treatments based on bioactive edible coating, ozonation, and gamma irradiation on ready-to-eat frozen green peppers: Evaluation of their freshness and sensory qualities. Eur. Food Res. Technol..

[138] Lima G.P.P., Machado T.M., Oliveira L.M., Silva Borges L., Pedrosa V.A., Vanzani P., Vianello F. (2014). Ozonated water and chlorine effects on the antioxidant properties of organic and conventional broccoli during postharvest. Sci. Agric..

[139] Chauhan O.P., Raju P.S., Ravi N., Singh A., Bawa A.S. (2011). Effectiveness of ozone in combination with controlled atmosphere on quality characteristics including lignification of carrot sticks. J. Food Eng..

[140] Hildebrand P.D., Forney C.F., Song J., Fan L., McRae K.B. (2008). Effect of a continuous low ozone exposure (50 nL L^-1^) on decay and quality of stored carrots. Postharvest Biol. Tec..

[141] De Souza L.P., Faroni L.R.A., Heleno F.F., Cecon P.R., Gonçalves T.C.G., da Silva G.J., Prates L.H.F. (2018). Effects of ozone treatment on postharvest carrot quality. LWT-Food Sci. Technol..

[142] Zhang L., Lu Z., Yu Z., Gao X. (2005). Preservation of fresh-cut celery by treatment of ozonated water. Food Control.

[143] Glowacz M., Rees D. (2016). Exposure to ozone reduces postharvest quality loss in red and green chilli peppers. Food Chem..

[144] Chitravathi K., Chauhan O.P., Raju P.S., Madhukar N. (2015). Efficacy of aqueous ozone and chlorine in combination with passive modified atmosphere packaging on the postharvest shelf-life extension of green chillies (*Capsicum annuum* L.). Food Bioproc. Technol..

[145] An J., Zhang M., Lu Q. (2007). Changes in some quality indexes in fresh-cut green asparagus pretreated with aqueous ozone and subsequent modified atmosphere packaging. J. Food Eng..

[146] Ölmez H., Akbas M.Y. (2009). Optimization of ozone treatment of fresh-cut green leaf lettuce. J. Food Eng..

[147] Akbas M.Y., Ölmez H. (2007). Effectiveness of organic acid, ozonated water and chlorine dippings on microbial reduction and storage quality of fresh-cut iceberg lettuce. J. Sci. Food Agric..

[148] Beltrán D., Selma M.V., Marín A., Gil M.I. (2005). Ozonated water extends the shelf life of fresh-cut lettuce. J. Agric. Food Chem..

[149] Hassenberg K., Idler C., Molloy E., Geyer M., Plöchl M., Barnes J. (2007). Use of ozone in a lettuce-washing process: An industrial trial. J. Sci. Food Agri..

[150] Gutiérrez D.R., Lemos L., Rodríguez S.C. (2018). Effect of UV-C and ozone on the bioactive compounds and antioxidant capacity of minimally processed rocket (*Eruca Sativa* Mill.). Int. J. New Technol. Res..

[151] Simão R., Neto D.G.T., Loose C.E. (2015). The ozonation as competitive advantage in post-harvest treatment of tomato. Mediterr. J. Soc. Sci..

[152] Onopiuk A., Półtorak A., Wojtasik-Kalinowska I., Szpicer A., Marcinkowska-Lesiak M., Pogorzelski G., Wierzbicka A. (2019). Impact of the storage atmosphere enriched with ozone on the quality of *Lycopersicon esculentum* tomatoes. J. Food Process. Preserv..

[153] Venta M.B., Broche S.S.C., Torres I.F., Perez M.G., Lorenzo E.V., Rodriguez Y.R., Cepero S.M. (2010). Ozone application for postharvest disinfection of tomatoes. Ozone Sci. Eng..

[154] Wang L., Fan X., Sokorai K., Sites J. (2019). Quality deterioration of grape tomato fruit during storage after treatments with gaseous ozone at conditions that significantly reduced populations of *Salmonella* on stem scar and smooth surface. Food Control.

[155] Tiwari B.K., O’Donnell C.P., Brunton N.P., Cullen P.J. (2009). Degradation kinetics of tomato juice quality parameters by ozonation. Int. J. Food Sci. Technol..

[156] Pignocchi C., Fletcher J.M., Wilkinson J.E., Barnes J.D., Foyer C.H. (2003). The function of ascorbate oxidase in tobacco. Plant Physiol..

[157] García-Viguera C., Bridle P. (1999). Influence of structure on colour stability of anthocyanins flavylium salts with ascorbic acid. Food Chem..

[158] Lee S.K., Kader A.A. (2000). Preharvest and postharvest factors influencing vitamin C content of horticultural crops. Postharvest Biol. Tec..

[159] Rabie M.A., Soliman A.Z., Diaconeasa Z.S., Constantin B. (2015). Effect of pasteurization and shelf life on the physicochemical properties of Physalis (*Physalis peruviana* L.) juice. J. Food Process. Preserv..

[160] Mirdehghan S.H., Valero D. (2017). Bioactive compounds in tomato fruit and its antioxidant activity as affected by incorporation of Aloe, eugenol, and thymol in fruit package during storage. Int. J. Food Prop..

[161] Szydłowska-Czerniak A., Trokowski K., Karlovits G., Szłyk E. (2010). Determination of antioxidant capacity, phenolic acids, and fatty acid composition of rapeseed varieties. J. Agric. Food Chem..

[162] Wold A.B., Rosenfeld H.J., Holte K., Baugerød H., Blomhoff R., Haffner K. (2004). Colour of post-harvest ripened and vine ripened tomatoes (*Lycopersicon esculentum* Mill.) as related to total antioxidant capacity and chemical composition. Int. J. Food Sci. Technol..

[163] Miller F.A., Silva C.L.M., Brandão T.R.S. (2013). A Review on ozone-based treatments for fruit and vegetables preservation. Food Eng. Rev..

[164] Sanoner P., Guyot S., Marnet N., Molle D., Drilleau J.F. (1999). Polyphenol profiles of French cider apple varieties (*Malus domestica* sp.). J. Agric. Food Chem..

[165] Bialka K.L., Demirci A. (2007). Decontamination of *Escherichia coli* O157: H7 and *Salmonella enterica* on blueberries using ozone and pulsed UV-light. J. Food Sci..

[166] Xu D., Shi M., Jia B., Yan Z., Gao L., Guan W., Wang Q., Zuo J. (2019). Effect of ozone on the activity of antioxidant and chlorophyll-degrading enzymes during postharvest storage of coriander (*Coriandrum sativum* L.). J. Process. Preserv..

[167] Xue J., Chen L., Wang H. (2008). Degradation mechanism of Alizarin Red in hybrid gas-liquid phase dielectric barrier discharge plasmas: Experimental and theoretical examination. Chem. Eng. J..

[168] Yeom H.W., Streaker C.B., Zhang Q.H., Min D.B. (2000). Effects of pulsed electric fields on the quality of orange juice and comparison with heat pasteurization. J. Agric. Food Chem..

[169] Rico D., Martin-Diana A.B., Frias J.M., Henehan G.T.M., Barry-Ryan C. (2006). Effect of ozone and calcium lactate treatments on browning and texture properties of fresh-cut lettuce. J. Sci. Food Agric..

[170] Ong M.K., Ali A., Alderson P.G., Forney F.C. (2014). Effect of different concentration of ozone on physiological changes associzted to gas exchange, fruit ripening, fruit surface quality and defence-related enzyme levels in papaya fruit during ambient storage. Sci. Hortic..

[171] Cullen P.J., Norton T., O’Donnell C., Tiwari B.K., Cullen P.J., Rice R.G. (2012). Ozone sanitisation in the food industry. Ozone in Food Processing.

